# Do quality of life, participation and environment of older adults differ according to level of activity?

**DOI:** 10.1186/1477-7525-6-30

**Published:** 2008-04-29

**Authors:** Mélanie Levasseur, Johanne Desrosiers, Denise St-Cyr Tribble

**Affiliations:** 1 Research Centre on Aging, Health and Social Services Centre – University Institute of Geriatrics of Sherbrooke (CSSS-IUGS), Sherbrooke, Québec, Canada; 2 Department of Rehabilitation, Faculty of Medicine and Health Sciences, Université de Sherbrooke, Sherbrooke, Québec, Canada; 3 Groupe de recherche interdisciplinaire en santé (Interdisciplinary Research Group on Health), Université de Montréal, Montréal, Québec, Canada; 4 University of Sherbrooke Affiliated Local Community Centre (CLSC component) of the CSSS-IUGS, Sherbrooke, Québec, Canada; 5 School of Nursing, Faculty of Medicine and Health Sciences, Université de Sherbrooke, Sherbrooke, Québec, Canada

## Abstract

**Background:**

Activity limitation is one of the most frequent geriatric clinical syndromes that have significant individual and societal impacts. People living with activity limitations might have fewer opportunities to be satisfied with life or experience happiness, which can have a negative effect on their quality of life. Participation and environment are also important modifiable variables that influence community living and are targeted by health interventions. However, little is known about how quality of life, participation and environment differ according to activity level. This study examines if quality of life, participation (level and satisfaction) and perceived quality of the environment (facilitators or obstacles in the physical or social environment) of community-dwelling older adults differ according to level of activity.

**Methods:**

A cross-sectional design was used with a convenience sample of 156 older adults (mean age = 73.7; 76.9% women), living at home and having good cognitive functions, recruited according to three levels of activity limitations (none, slight to moderate and moderate to severe). Quality of life was estimated with the Quality of Life Index, participation with the Assessment of Life Habits and environment with the Measure of the Quality of the Environment. Analysis of variance (ANOVA) or Welch F-ratio indicated if the main variables differed according to activity level.

**Results:**

Quality of life and satisfaction with participation were greater with a higher activity level (p < 0.001). However, these differences were clinically significant only between participants without activity limitations and those with moderate to severe activity limitations. When activity level was more limited, participation level was further restricted (p < 0.001) and the physical environment was perceived as having more obstacles (p < 0.001). No differences were observed for facilitators in the physical and social environment or for obstacles in the social environment.

**Conclusion:**

This study suggests that older adults' participation level and obstacles in the physical environment differ according to level of activity. Quality of life and satisfaction with participation also differ but only when activity level is sufficiently disrupted. The study suggests the importance of looking beyond activity when helping older adults live in the community.

## Background

Aging of the population, reform of the health care system and individual preferences increase the number of older adults with a decline in functional independence who live in the community. A decline in functional independence, or activity limitations according to the terminology of the International Classification of Functioning, Disability and Health (ICF) [1], is one of the most frequent geriatric clinical syndromes that have significant individual and societal impacts [2]. People living with activity limitations might have fewer opportunities to be satisfied with life or experience happiness, which can have a negative effect on their quality of life (QOL) [3]. Quality of life may be defined as the sum of cognitive and emotional reactions that an individual experiences associated with his/her achievements [4] in the context of his/her culture and values, taking into account his/her goals, expectations, standards, and concerns [5]. This definition has the advantage of partially including one of the most cited QOL definitions developed by the World Health Organization Quality of Life (WHOQOL) Group and has been modified to address criticism about its lack of emphasis on the individual's reactions. As improving or maintaining QOL is the ultimate goal of health interventions [6-8], it is important to have a better understanding of the QOL of older adults with different activity levels.

**Figure 1:**
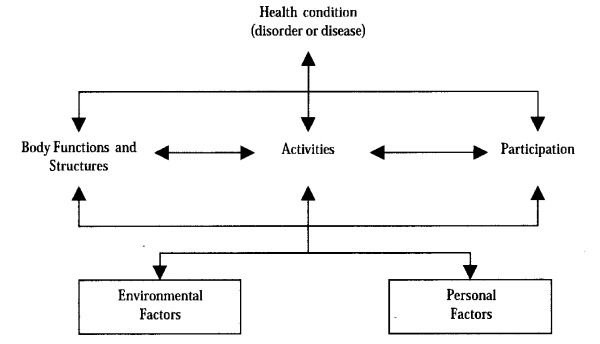
**International Classification of Functioning, Disability and Health (ICF) model**. Taken from: World Health Organization (WHO) (2001). International Classification of Functioning, Disability and Health. Geneva, Switzerland: WHO.

Participation and environment are also important modifiable variables influencing community living ([9] and targeted by health interventions [10-13]. Like activity level, they are components of the ICF. According to the ICF (Figure 1), environmental factors include the physical, social and attitudinal environment in which people live and conduct their lives [1]. Participation is the result of interaction between the individual's health and contextual factors that include both personal and environmental factors. While activity is defined as an individual's ability to perform a task or action, participation is defined as involvement in a life situation [1] including accomplishment of daily activities and social roles [9]. For example, the capacity to walk 100 feet refers to activity whereas walking in one's environment while doing daily activities refers to participation. Satisfaction with participation is closely related to personal goals and priorities [4] and might better reflect an individual's perception of his/her optimal participation level [14]. The concept of activity is central to the ICF (Figure 1) and was traditionally considered one of the key outcomes in successful community living [15]. However, little is known about how QOL, participation and environment differ according to activity level. Since it is increasingly recognized that body functions and structures are more intrinsically linked to activity level, they were not considered in this study.

From a theoretical viewpoint, it is reasonable to assume that QOL decreases with activity limitations [2]. However, previous studies with older adults have produced inconsistent findings: some supported the importance of activity for QOL [2,16-23] while others showed limited influence [16,24,25]. A narrow range of activity level of participants or absence of comparison groups without activity limitations as well as the lack of an underlying conceptual model, however, limit the strength of the conclusions of most of these studies.

Furthermore, recent theory also shaped QOL studies. According to response shift theory [26-28], the meaning of one's QOL self-evaluation might change over time and is not linear, allowing the person to maintain an equilibrium in his/her QOL assessment. Response shift is usually initiated by a change in health that may affect the person's activity level and can result in changes in his/her internal standards, changes in the importance of values, or reconceptualization of QOL [26-28]. With these changes, the person might give less importance to some aspects such as health and functioning and more to others like family or spirituality. This new way to evaluate QOL might generate the same global appreciation despite the presence of a health problem. Therefore, studies on QOL should consider response shift [26-28], which may threaten the validity of research assumptions and therefore the foundation of self-reported QOL measures [29].

Previous studies and clinical interventions mostly targeted activity level [15]. However, there is increasing evidence that participation embraces the complexity of human functioning better [1] and goes beyond activity level [30]. Participation has been shown to decrease in normal aging [31], be more restricted by disabilities in old age [23] and not be totally explained by activity level [30-32] Well-adapted individuals might be satisfied with their participation level even if it is restricted [33,34].

Although the importance of environmental factors was considered in the ICF, there is little evidence that supports its inclusion [35,36]. There is a need for more knowledge about how elderly people with disability perceive their environmental factors to influence their participation [37]. For individuals with activity limitations, support from the social environment [38,39] and accessibility of the physical environment [1,33,39-41] may be seen as imperatives to help them live in the community [37,42]. However, results of previous studies are not consistent regarding the beneficial effect of social support on activity level [43], and little research has been done to document whether the environment has an influence on the activity level of older adults [43,44]. Individuals' perceptions of both the physical and social environment might differ according to their level of activity. Because of different life experiences, some aspects of the environment are perceived as a facilitator or an obstacle to their participation. It is important to better understand the impact of environmental factors. In fact, these factors can directly increase the risk of activity limitations or exacerbate the negative impact of other personal risk factors [45]. Interventions targeting the environment may have a greater impact on an individual's activity level than those targeting individuals factors [46].

From this perspective, the present study aimed to explore, based on the ICF, if QOL, participation and environment of adults aged sixty and over differ according to their level of activity.

## Methods

### Participants

This cross-sectional design involved 156 persons with different activity levels, aged 60 and over, and living in the community. Eligibility criteria were: 1) good cognitive functions (score on the Mini-Mental State Examination [47] equal to or above the 25th percentile for age and schooling [48]); 2) good understanding of French or English; and 3) a level of activity corresponding to one of the three equal-sized groups created accordingly, as measured by the Functional Autonomy Measurement System (SMAF) [49]. The SMAF includes 29 functions covering 5 domains (number of items): activities of daily living (7), mobility (6), communication (3), mental functions (5), and instrumental activities of daily living (8). Each function is scored on a 5-point scale: 0 (independent), 0.5 (difficulty), 1 (needs supervision), 2 (needs help), 3 (dependent). The psychometric properties were studied with older adults and are good: high intraclass correlation coefficients (ICC) for test-retest (0.95) and interrater (0.75) reliability and good discriminant validity [50]. For the first group (G1), participants needed to have a score < 5, suggesting a good activity level; for the second group (G2), a score between 5 and 19, indicating slight to moderate activity limitations; and, for the third group (G3), a score > 19, suggesting moderate to severe activity limitations. These cut-off scores were used in other studies [51,52], considered the potential measurement error of 5 points, and were discussed with the authors of the tool and based on many years of clinical observations. At the time of their recruitment, participants with activity limitations were receiving services from a local community service centre, geriatric day hospital or geriatric day centre, the recruitment sites of the study. Participants without activity limitations were recruited from a previous study on healthy aging. People were excluded if they were terminally ill or had moderate to severe language deficits. This study was approved by the Research Ethics Committees of the University Institute of Geriatrics of Sherbrooke and the Eastern Townships Multivocational Institutions providing Home and Community Services.

### Data collection procedures

All participants who were eligible, until the predetermined sample size (n = 52 per group) was reached, signed an informed consent form and were evaluated in about 90 minutes at their homes by one of the three occupational therapists specifically trained to administer the questionnaires. The usual sociodemographic and clinical data (see Table 1), mostly associated with the personal factors of the ICF, were collected first. The International Classification of Diseases (ICD-10) [53] was used to identify the disease category that best represented the health condition of each participant. Comorbidity was measured with the Charlson Index [54], which includes 30 conditions rated on a four-level Likert scale. Three questionnaires concerning the individual's perceptions were used to collect data on the main variables: QOL, participation and environment.

**Table 1:** Characteristics of the participants (n = 52 per group)

Continuous variables	G1 Mean (SD)	G2 Mean (SD)	G3 Mean (SD)	p value
Functional independence (SMAF;/87)	1.4 (1.6) ^a^	10.2 (4.1) ^b^	29.0 (7.6)	< 0.001^c^
Age (years)	70.2 (7.0) ^a^	74.9 (8.4)	75.8 (7.6)	< 0.001^d^
Associated conditions (#)	0.8 (1.2) ^a^	1.5 (1.4) ^b^	3.3 (2.3)	< 0.001^d^

Categorical variables	Frequency (%)	Frequency (%)	Frequency (%)	

Gender (women)	40 (76.9) ^a^	28 (53.8)	26 (50)	0.01^e^

Education (years):				
- 1–6	4 (7.7)	14 (26.9)	14 (26.9)	0.28^d^
- 7–11	27 (51.9)	23 (44.2)	23 (44.2)	
- 12–14	18 (34.6)	8 (15.4)	9 (17.3)	
- > 15	3 (5.7)	7 (13.5)	6 (11.6)	

Residential status:				
- Owner	29 (55.8) ^f^	24 (46.2)	16 (30.8)	0.04^e^
- Tenant	22 (42.3)	22 (42.3)	20 (38.5)	
- Other	1 (1.9)	6 (11.5)	16 (30.8)	

Income (Can $):				
- < 15,000	11 (21.2)^a^	21 (40.4)	19 (36.5)	0.006^d^
- 15,001- 25,000	9 (17.3)	13 (25.0)	14 (26.9)	
- > 25,001	21 (40.4)	16 (30.8)	15 (28.8)	
*Missing Data*	11 (21.2)	2 (3.8)	4 (7.7)	

Classification of diseases (ICD-10):				
- Diseases of the nervous system	1 (1.9) ^a^	5 (9.6) ^b^	22 (42.3)	< 0.001^e^
- Diseases of the circulatory system	26 (50.0)	17 (32.7)	7 (13.5)	
- Injury, poisoning and certain other consequences of external causes (including hip fracture)	1 (1.9)	12 (23.1)	9 (17.3)	
- Diseases of the musculoskeletal system and connective tissue	7 (13.5)	12 (23.1)	8 (15.4)	
- Other	17 (32.7)	6 (11.5)	6 (11.5)	

Self-perceived health:				
- Excellent	27 (51.9) ^a^	10 (19.2) ^b^	1 (1.9)	< 0.001^c^
- Good	21 (40.4)	25 (48.1)	16 (30.8)	
- Fair	4 (7.7)	14 (26.9)	26 (50.0)	
- Poor	0 (0)	3 (5.8)	9 (17.3)	

Stability of self-perceived capacities (Yes)	52 (100.0) ^a^	39 (75.0)	42 (80.8)	0.001 e
Self-perceived mood (not depressed)	48 (92.3) ^f^	42 (80.8)	35 (67.3)	0.006^e^

G1: SMAF < 5 G2: SMAF [5, 19] G3: SMAF > 19 ICD-10: International Classification of Diseases

^a ^: G1 differs significantly from the other two groups on these variables (p < 0.017).

^b ^: G2 differs significantly from G3 on these variables (p < 0.017).

^c ^: p value associated with ANOVA. A significant value (p < 0.05) indicates a difference between the three groups.

^d ^: p value associated with Welch F-ratio. A significant value (p < 0.05) indicates a difference between the three groups.

^e^: χ^2 ^test

^f ^: G1 differs significantly only from G3 on these variables (p < 0.017).

### Measurement instruments

Quality of life was estimated with the Quality of Life Index (QLI) [55], which is a generic satisfaction with life tool that takes the individual's reactions into account [56]. It includes 32 items related to four life domains (number of items): Health and functioning (11), Socio-economic (10), Psychological/spiritual (7) and Family (4). Each item is evaluated by the participant on two 6-point Likert scales ranging from 'very dissatisfied' (1) to 'very satisfied' (6) or 'not important' (1) to 'very important' (6). The importance scores allow weighting of the satisfaction scores, reflecting both the individual's satisfaction and importance of values. This importance score can be used to partially assess response shift. The scale ranges from 0 to 30 for each domain and for the total score, with scores of 19 or less indicating poorer QOL (tool and details about scoring available at [57]). The total score normal range is 23.0 (SD = 4.0) and a difference of 2–3 points represents a change that is noticeable in practice, i.e. is clinically meaningful [58]. The internal consistency of the QLI is supported by several studies (Cronbach's alphas = 0.76 to 0.91) [21,25]. Good test-retest reliability (r = 0.81 to 0.87) and concurrent validity with one measure of life satisfaction (r = 0.65 to 0.75) have also been demonstrated [55].

The Assessment of Life Habits (Life-H) short 3.0 version [59] is a questionnaire assessing level of accomplishment in daily activities and social roles (participation), and satisfaction with this accomplishment level (satisfaction with participation). The Life-H 3.0 is composed of 69 items divided into 12 domains of life. These domains (number of items) are: nutrition (3), fitness (3), personal care (7), communication (7), housing (8), mobility (5), responsibilities (6), interpersonal relationships (7), community life (7), education (3), employment (7) and recreation (6). The first six domains refer to daily activities while the other six are associated with social roles. Participation is based on the level of difficulty and assistance used to carry out the activities or roles, and ranges from 0 (not accomplished) to 9 (accomplished without difficulty). Normal range scores are 8.1 (SD = 0.5) for daily activities and 8.2 (SD = 0.8) for social roles [30] and a change of 0.5 is clinically significant [60]. Satisfaction with each item is rated on a 5-point Likert scale ranging from 1 (very dissatisfied) to 5 (very satisfied). Two scores are reported for both level of and satisfaction with participation: the mean subscore for daily activities and the mean subscore for social roles. The psychometric properties of the level of participation scale, studied with older adults, are good: high global ICC for test-retest (0.95) and interrater (0.89) reliability for the total score [61] and good construct validity [30].

The Measure of the Quality of the Environment (MQE) version 2.0 [62] documented the self-perceived physical and social environment, i.e., whether each environmental item is perceived as a facilitator or an obstacle in the accomplishment of daily activities and social roles. The MQE comprises six domains which cover most aspects of the environment (number of items): social support and attitudes (14), income, labour and income security (15), government and public services (27), equal opportunities and political orientations (10), physical environment and accessibility (38), and technology (5). Generally, the last two domains refer to the physical environment (40 items) while the rest refer to the social environment (69 items). The person's perception is rated on a 7-point Likert scale ranging from -3 (major obstacle) to 3 (major facilitator), allowing weighting of the items. As Whiteneck and colleagues [35] indicated that insurmountable barriers which are systematically avoided may not be reported per se, the interviewer (occupational therapist) further questioned the person when the rating might not have fully considered the reality. Two continuous scores, an "obstacle" score and a "facilitator" score, are calculated by summing the weighted items for both the physical and social environments. The mean number of items perceived as facilitators or obstacles out of 40 (physical environment) or 69 (social environment) is also reported. A test-retest reliability study showed moderate to high kappas for 57% of the items [63].

### Statistical analysis

Characteristics of the participants were described by means and standard deviations or frequencies and percentages according to the type of variable (continuous or categorical, respectively) and compared across the groups with the chi square test (dichotomized categories) or analysis of variance (ANOVA). Chi square and t tests also compared the sociodemographic characteristics of participants with those who refused to participate. When homogeneity of variance was not respected, the Welch F-ratio was calculated instead of ANOVA.

The mean score (out of 6) was calculated using the QLI "satisfaction" and "importance" scores. ANOVA or Welch F-ratio was then used to determine whether QLI satisfaction and importance differed depending on the level of activity. These tests also indicated if the main variables differed according to activity level. When statistical differences were identified, two-by-two tests (multiple comparisons) were calculated to locate the differences, with a p value of 0.017 (Bonferroni's correction).

Regression analyses were also performed to identify whether QLI, Life-H and MQE differences between the groups persisted when controlling for confounding variables. These confounding variables differed between the groups and were associated with the corresponding main variable.

## Results

Fifty-two participants per activity level group were recruited. A total of 198 people were contacted in order to obtain the predetermined sample size. Those who refused to participate (n = 42) were older and had less schooling and a lower income than those who agreed. The sociodemographic characteristics of participants are presented and compared in Table 1. Participants with no activity limitations (G1) were younger and mostly female. Compared to G1, fewer G3 participants lived in their own home and more of them had a lower income as well as perceived themselves as depressed.

Generally, the QLI scores were significantly lower with more activity limitations, except for the "Family" domain which also obtained the highest mean scores (Table 2). The G3 "Health and functioning" domain was the only QLI score below 19, indicating poorer QOL. The QLI total score varies by nearly 2 points between each group. When controlled for the confounding variables (income, residential status and self-perceived mood), the difference between the groups' QLI total score persisted by 1.2 points out of 30 between each group (p < 0.001). This difference is clinically significant only between G1 and G3.

**Table 2:** Comparisons of scores on main variables by group (n = 52 per group)

Continuous variables	G1 Mean (SD)	G2 Mean (SD)	G3 Mean (SD)	p value
**1. Quality of life (QLI;/30)**				
- Health and functioning	23.2 (2.7) ^a^	20.1 (4.0) ^b^	16.5 (3.8)	< 0.001^c^
- Socio-economic	23.1 (2.8) ^a^	21.2 (3.3)	20.3 (3.5)	< 0.001^c^
- Psychological/spiritual	23.4 (2.4) ^d^	22.8 (3.7)	21.6 (3.3)	0.009^e^
- Family	23.6 (4.0)	23.7 (5.2)	23.2 (4.0)	0.83^c^
Total score	23.3 (2.3) ^a^	21.5 (3.1) ^b^	19.6 (2.9)	< 0.001^c^

**2. Participation (Life**-**H)**				
• Accomplishment scale (/9)				
- Daily activities	8.3 (0.4) ^a^	7.3 (0.7) ^b^	5.4 (0.9)	< 0.001^e^
- Social roles	8.6 (0.6) ^a^	7.1 (1.4) ^b^	5.1 (1.1)	< 0.001^e^
• Satisfaction scale (/5)				
- Daily activities	4.2 (0.3) ^a^	4.0 (0.4) ^b^	3.5 (0.4)	< 0.001^c^
- Social roles	4.2 (0.3) ^a^	4.0 (0.4) ^b^	3.6 (0.5)	< 0.001^c^

**3. Environment (MQE)**				
• Facilitators				
- Physical (# of items;/40)	21.3 (8.1) ^f^	25.3 (5.4) ^b^	22.3 (5.1)	0.003^e^
Weighted number	50.8 (22.0)	56.5 (12.1) ^b^	49.2 (11.9)	0.009^e^
- Social (# of items;/69)	29.6 (7.8)	32.3 (6.2)	30.4 (6.4)	0.13^c^
Weighted number	67.3 (20.3)	68.8 (14.7)	66.6 (17.4)	0.77^e^
• Obstacles				
- Physical (# of items;/40)	6.8 (3.9) ^a^	9.0 (4.3)	10.9 (4.0)	< 0.001^c^
Weighted number	11.5 (8.3) ^a^	17.0 (10.1) ^b^	22.7 (10.6)	< 0.001^e^
- Social (# of items;/69)	1.4 (1.3)	2.3 (3.3)	2.5 (3.0)	0.03^e^
Weighted number	2.3 (2.4) ^d^	4.5 (7.3)	4.8 (6.8)	0.009^e^

G1: SMAF < 5 G2: SMAF [5, 19] G3: SMAF > 19

^a ^: G1 differs significantly from the other two groups on these variables (p < 0.017).

^b ^: G2 differs significantly from G3 on these variables (p < 0.017).

^c ^: p value associated with ANOVA. A significant value (p < 0.05) indicates a difference between the three groups.

^d ^: G1 differs significantly only from G3 on these variables (p < 0.017).

^e ^: p value associated with Welch F-ratio. A significant value (p < 0.05) indicates a difference between the three groups.

^f ^: G1 differs significantly only from G2 on this variable (p < 0.017).

QLI: Quality of Life Index (normal range = 23.0; SD = 4.0) Life-H: Assessment of Life Habits (normal range for daily activities = 8.1; SD = 0.5 and for social roles = 8.2; SD = 0.8) MQE: Measure of the Quality of the Environment

The participants' QLI satisfaction and importance scores also decreased significantly across the groups for the total score and each of the life domains, except the "Family" domain (Table 3). The importance score of the "Psychological/Spiritual" domain and the total score were also similar in each group and high. "Health and functioning" was the QLI domain that mainly differed between the groups, especially for the satisfaction score, which appears to be the only one that is clinically significant.

**Table 3:** Comparisons of satisfaction and importance scores of quality of life index by group (n = 52 per group)

Continuous variables (/6)	G1 Mean (SD)	G2 Mean (SD)	G3 Mean (SD)	p value
Health and functioning:				
- Satisfaction	5.0 (0.5)	4.4 (0.8)	3.8 (0.8)	< 0.001^a^
- Importance	5.1 (0.5)	5.1 (0.5)	4.8 (0.6)	0.01^b^
Socio-economic:				
- Satisfaction	4.6 (0.5)	4.3 (0.6)	4.1 (0.6)	< 0.001^b^
- Importance	4.5 (0.4)	4.4 (0.4)	4.3 (0.6)	0.045^a^
Psychological/spiritual:				
- Satisfaction	5.1 (0.4)	4.9 (0.6)	4.7 (0.6)	0.003^a^
- Importance	5.3 (0.4)	5.3 (0.5)	5.2 (0.7)	0.51^b^
Family:				
- Satisfaction	4.7 (0.9)	4.8 (1.1)	4.6 (0.9)	0.60^b^
- Importance	5.4 (0.8)	5.6 (0.6)	5.4 (0.8)	0.48^b^
Total score:				
- Satisfaction	4.8 (0.4)	4.5 (0.6)	4.2 (0.6)	< 0.001^b^
- Importance	5.0 (0.3)	5.0 (0.3)	4.8 (0.5)	0.07^a^

G1: SMAF < 5 G2: SMAF [5, 19] G3: SMAF > 19

^a ^: p value associated with Welch F-ratio. A significant value (p < 0.05) indicates a difference between the three groups.

^b ^: p value associated with ANOVA. A significant value (p < 0.05) indicates a difference between the three groups.

Level of participation also decreased between each group for both daily activities and social roles, but the difference was greater between G2 and G3 than between G1 and G2 (Table 2). Even after controlling for age, income and self-perceived mood, the differences between each group persisted, with scores decreasing by 1.3 (daily activities) or 1.5 (social roles) out of 9 (p < 0.001), and were clinically significant.

Satisfaction with participation scores was also lower with additional activity limitations between each group for both daily activities and social roles (Table 2). Again, compared to the difference between G1 and G2, the greatest difference was found between the two groups with activity limitations (G2 and G3). These differences persisted after controlling for age and self-perceived mood, decreasing by 0.3 (daily activities) or 0.2 (social roles) points out of 5 (p < 0.001), and appear to be clinically significant only between G1 and G3.

Generally, the environment was mainly perceived as a facilitator in the accomplishment of daily activities and social roles while obstacles in the environment were primarily attributed to the physical environment (Table 2). Between-group differences were observed for facilitators in the physical environment as well as for obstacles in the physical and social environment. However, after controlling for income and residential status, differences according to level of activity persisted only for obstacles in the physical environment (difference of 5.1 points for the weighted items between each group (p < 0.001)).

Participants with activity limitations (G2 and G3) did not differ in their perceived number of obstacles in the physical environment, but these obstacles, as measured by the MQE, seemed to disrupt G3's participation more (Table 2). Group 1 and G3 participants did not differ in their perceived number of obstacles in the social environment, but these obstacles appeared to affect participation more in G3 than G1. Finally, G2 participants perceived more facilitators in their physical environment than G1, but these facilitators seem to not affect participation differently in these two groups.

## Discussion

The main objective of this study was to examine QOL, participation and environment according to older adults' level of activity. The results showed that QOL decreased according to activity limitations, suggesting that a reduced activity level is associated with decreased QOL. However, QOL diminished only slightly across the groups, after controlling for income, residential status and mood, and were clinically significant only between participants without activity limitations and those with moderate to severe activity limitations. Moreover, except for the G3 "Health and functioning" domain, QLI scores were not low enough to qualify as poor QOL. These high QOL scores suggest that participants modified their assessment of their QOL, i.e. underwent a response shift [26-28]. Other studies discovered that it is difficult to live with decreased QOL [34], with many people living with significant and persistent activity limitations reporting good or excellent QOL [33,34,64]. As suggested by two qualitative studies with adults having activity limitations [33,34], adaptation appears to have more influence on QOL than activity limitations by themselves. Finally, QOL of polio survivors was found to be similar, regardless of the severity of symptoms, but lower than that of healthy people, mainly for the health domain [65].

As expected, because of the activity level recruitment criteria, the greatest differences between the groups was in QLI satisfaction scores in the "Health and functioning" domain. Also because of the activity level recruitment criteria, we expected to find a response shift. This could be initiated by a change in internal standards (approximately the same level of satisfaction on the QLI between groups) or a change in values (a difference in the importance score of the QLI between groups). In fact, based on the QLI importance scores, between-group differences were small. The change in internal standards or values proposed by the response shift theory was therefore only partially supported by our data. However, response shift can also result from reconceptualization of QOL [26-28], and this was not taken into account in our study. In addition, the QOL comparisons were not on the same individuals (longitudinal), making response shift considerations only exploratory.

As expected and consistent with the ICF, level of participation decreased with increased activity limitations, as supported by other studies [30,66,67]. Furthermore, in a study with people who had a stroke [32], age together with level of impairment and disability explained a substantial part, 53%, of the variance in participation. Another cross-sectional study, this time with people with spinal cord injury, found [35], demonstrated that restricted participation is best explained (20%) by more limitations in activity. In our study, G1 participation scores very similar to those obtained in a study on normal aging [30] and G3 scores were similar to people who had a stroke [60]. However, as participation had been previously demonstrated to go beyond activity level [30], our results highlight the importance of differentiating better between the operationalization of activity and participation as proposed by the ICF.

To our knowledge, variations in satisfaction with participation according to older adults' activity level have not been previously documented. Satisfaction with participation might represent older adults' adaptation and selection of activities that are most important to them. In the present study, satisfaction with participation decreased according to level of activity but was clinically significant only between participants without activity limitations and those with moderate to severe activity limitations. Like QOL, satisfaction can be modified by a response shift. It is not clear that a response shift occurred here in regard to satisfaction with participation since neither the participation measurement tool nor the study design allowed full consideration of the response shift.

Self-perceived depressed mood differs according to activity level, QOL and level of and satisfaction with participation. Older adults with depressed mood may do fewer activities and restrict their participation, which in turn may influence their QOL. However, this cross-sectional study did not allow us to clarify if depressed mood causes a lower activity level, restriction in participation or lower QOL.

Even if theoretically the social and physical environment can facilitate or impede participation, the role of environmental factors in human functioning is not as simple. In this study, perceived obstacles in the physical environment increased according to activity level and seem to affect the participation of older adults having moderate to severe activity limitations more than those with slight to moderate limitations. Obviously, people having greater difficulty walking and moving around find the physical environment less user-friendly. In fact, two studies showed that many people with disability feel estranged and oppressed by facets of the built environment [68] and that subjects with more activity limitations reported more barriers [35]. An adaptive environment is a salient feature for people with physical disabilities [69]. However, a recent study with older adults showed that physical barriers were not an important issue for participation because of help from the social environment [37]. In addition, perceived obstacles in the social environment increased between G1 and G3. Social support and attitudes might be seen as not or less helpful for people with activity limitations. These people often have limited income and considerable expenses associated with their health problem [70] and might perceive public and government services as less adapted to their specific needs. However, familiarity and lifelong experience can also influence individuals' perception of their environment.

Environments that present more barriers and fewer resources might trigger a pattern of disuse and subsequent reductions in activity level, speeding up the aging process [39]. Older adults with activity limitations have been known to experience an increased sensitivity to physical barriers in the environment [38]. Another longitudinal study with older adults showed that living in a deficient environment was associated with an increased risk of overall activity loss [44]. However, this populational study focused on a small number of negative environmental characteristics and did not use a standardized instrument to measure their participants' activity level. Finally, as postulated in a study with people with spinal cord injury [35], people facing barriers may, with added difficulty, be able to overcome them (participation) but that the experience of encountering barriers may reduce QOL.

Surprisingly, facilitators in the social environment were not perceived differently by the groups. Rochette and collaborators [32] found that facilitators in the environment are not associated with participation. Since the importance of social support for people with activity limitations has been documented by many studies [24,25,33,64,71] and community resources and services are usually not sufficient, older adults with activity limitations might need further help from their social environment. When desired by the person, social support such as encouraging, supportive family and friends would be extremely valuable in counteracting obstacles and enhancing health and QOL [72].

Increasing older adults' activity level or facilitators in their environment and reducing obstacles in their environment can mainly be achieved by proper coordination of health services. Older adults' health programs and strategies traditionally target personal factors to the detriment of environmental factors that favor health and activities [73]. Prevention programs and new government policies are also necessary to increase facilitators and lessen obstacles in the environment. For example, a prevention program can increase social support or government policies can favour implementing age-friendly cities advocated by the World Health Organisation (WHO) to promote older adults' participation. Environmental factors need to support and reinforce older adults' competence, facilitate adaptation, and compensate for activity limitations [39].

### Study limitations and strengths

This study was carried out with a convenience sample of people having good cognitive functions and, for those with activity limitations, receiving health or community services that may positively influence their QOL, and might not be fully representative of older adults having activity limitations and living in the community. The comparison between the main variables was cross-sectional but the sample size was sufficient (n = 52) to allow detection of a standardized difference smaller than 0.4 between two means for a p value of 0.05 and power at 80% [74]. Finally, some items of the measurement tools were similar and might partly explain some differences between the groups, especially for participation level.

Nevertheless, this study is a first step in understanding variations in QOL, participation and environment according to the activity level of older adults. The strengths of the study are the creation of groups based on activity level to address the research objective, the underlying conceptual model (ICF), the consideration of important modifiable variables targeted by health interventions, and the rigorous methodology including validated tools.

## Conclusion

This study demonstrated that older adults' QOL and satisfaction with participation vary according to activity level, but mainly when the latter is sufficiently disrupted. Level of participation and perceived obstacles in the environment also vary with level of activity. Finally, the study suggests the importance of looking beyond activity measures to help community-living older adults with activity limitations.

## List of abbreviations used

ANOVA: Analysis of variance; CIHR: Canadian Institutes of Health Research; FRSQ: Fonds de la recherche en santé du Québec; G1: First group, participants with a SMAF score < 5, suggesting a good activity level; G2: Second group, participants with a SMAF between 5 and 19, indicating slight to moderate activity limitations; G3: Third group, participants with a SMAF score > 19, suggesting moderate to severe activity limitations; ICC: Intraclass correlation coefficients; ICD-10: International Classification of Diseases; ICF: International Classification of Functioning, Disability and Health; Life-H: Assessment of Life Habits; MQE: Measure of the Quality of the Environment; SMAF: Functional Autonomy Measurement System; QLI: Quality of Life Index; QOL: Quality of life.

## Competing interests

The authors declare that they have no competing interests.

## Authors' contributions

ML conceived the study, participated in the data collection, coordinated the study, performed the statistical analysis and drafted the manuscript. JD and DST participated in the design and helped to draft the manuscript. All authors read and approved the final manuscript.
